# Prostatic Artery Embolization for the Treatment of Benign Prostate Hyperplasia: Initial Experience From Bahrain

**DOI:** 10.7759/cureus.22593

**Published:** 2022-02-25

**Authors:** Wael Hamed Ibrahim, Hiba Abduljawad, Hosameldin Mohamed, Noora Jamsheer, Mohamed Elsayed Elnaggar

**Affiliations:** 1 Medical Imaging, King Hamad University Hospital, Muharraq, BHR; 2 Radiology, King Hamad University Hospital, Muharraq, BHR

**Keywords:** bahrain, embolization, prostate, prostatic artery, benign prostate hyperplasia

## Abstract

Introduction: Benign prostate hyperplasia (BPH) is a common problem in elderly men. The current gold standard is surgical transurethral resection of the prostate (TURP). Prostatic artery embolization (PAE) is an alternative treatment of BPH, which avoids surgical complications. This is a single-center prospective study from the Kingdom of Bahrain to evaluate the effectiveness of PAE.

Methods: This prospective, single-center study included consecutive patients eligible for PAE. Patients were evaluated at one, three, six, and 12 months. Clinical success was defined as a decrease of International Prostate Symptom Score (IPSS) ≥ 3 points, quality of life (QoL) score ≤ 3 or decrease by three points, and no need for surgical intervention. Correlation between prostate-specific antigen (PSA) at 24 hours and reduction in prostate volume and IPSS score was also assessed.

Results: A total of 29 patients underwent the procedure between June 2015 and August 2018. Bilateral embolization was achieved in 26 patients. Clinical success was achieved in 26 patients (89.65%). No major adverse events were encountered. Significant improvement for storage (−4.54 ± 2.93, p < 0.005) and voiding symptoms score (−7.54 ± 4.74, p <0.005) were seen. There was no significant correlation between 24 hours PSA and reduction of the size of prostate and IPSS score at 12 months.

Conclusion: PAE is a minimally invasive procedure that is safe and effective for the management of BPH. PAE is effective for the management of both storage and voiding symptoms.

## Introduction

Benign prostate hyperplasia (BPH) is a common problem in elderly men. Patients present with lower urinary tract symptoms in the form of obstructive and storage symptoms. Over 30% of men above the age of 50 years suffer from lower urinary tract symptoms suggestive of BPH [1]. Transurethral resection of the prostate (TURP) is the current gold standard for the treatment of BPH. It has excellent clinical outcome; however, the associated risks are what makes prostatic artery embolization (PAE) a feasible alternative. TURP has intra- and post-operative complications such as bleeding, incontinence, sexual dysfunction, and dilutional hyponatremia [2]. PAE is currently a well-established technique with promising published data [3]. This is a single-center prospective study from the Kingdom of Bahrain.

## Materials and methods

Population

Protocols applied for this prospective study have been approved by the Research and Ethics Committee of King Hamad University Hospital. Patients with moderate to severe lower urinary tract symptoms (International Prostate Symptom Score (IPSS) ≥ 8) and no symptomatic improvement on medical treatment alone for a minimum of six months were included in the study. Exclusion criteria were allergy to iodinated contrast, evidence of prostate malignancy, and patients with neurogenic bladder proved by cystometry study if available.

Prior to scheduling for the procedure, the patients provided written consent and were then asked to attend the radiology department for pre-procedural evaluation. The evaluation included answering the questionnaires of the IPSS, quality of life (QOL) questionnaire, and International Index of Erectile Function (IIEF). Blood tests included prostate-specific antigen (PSA), complete blood count (CBC), renal function, urinalysis, and coagulation profile. All patients underwent MRI prostate and flowmetry prior to the procedure.

Procedure

**Figure 1:**
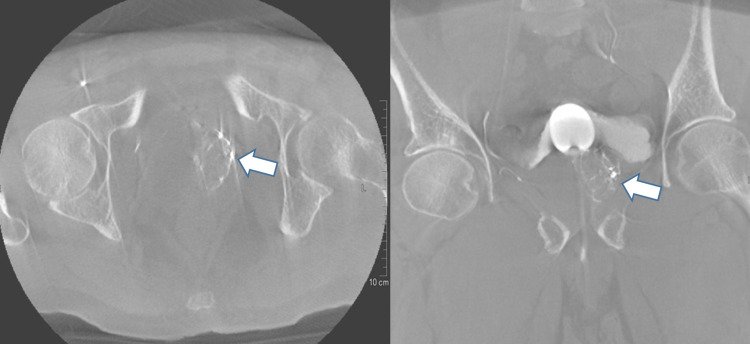
Cone beam CT (axial image, left; coronal reconstruction, right) done from microcatheter to ensure the proper position of microcatheter before embolization, showing proper opacification of the transition zone at the left side (white arrow), with no detectable opacified extra-prostatic branches confirming proper site for embolization.

All PAE procedures were performed by one consultant (WI). Siemens Artis (Siemens Healthineers, Erlangen, Germany) ceiling angiography suite was used. A Foley catheter was inserted in the bladder and was filled with contrast (50% visipaque mixed with saline), to identify the level of the prostatic artery. Under aseptic technique and local anesthesia, a right common femoral artery access was secured by 5 F sheath, through which 5 F pigtail catheter was manipulated over a guidewire and placed at the distal abdominal aorta. A pelvic angiogram was done to clearly delineate both prostatic arteries. Coaxial selective cannulation of the left prostatic artery was done using a headway 16 Duo microcatheter. Cone beam CT (CBCT) was used for confirming the position of the prostatic artery and sites of anastomoses (Figure 1). A protective coil was used when there was a risk of non-target embolization by a point of anastomosis. Direct intra-arterial injection up to a total amount of 400 microgram nitroglycerine through the microcatheter was injected into both prostatic arteries prior to embolization.

**Figure 2:**
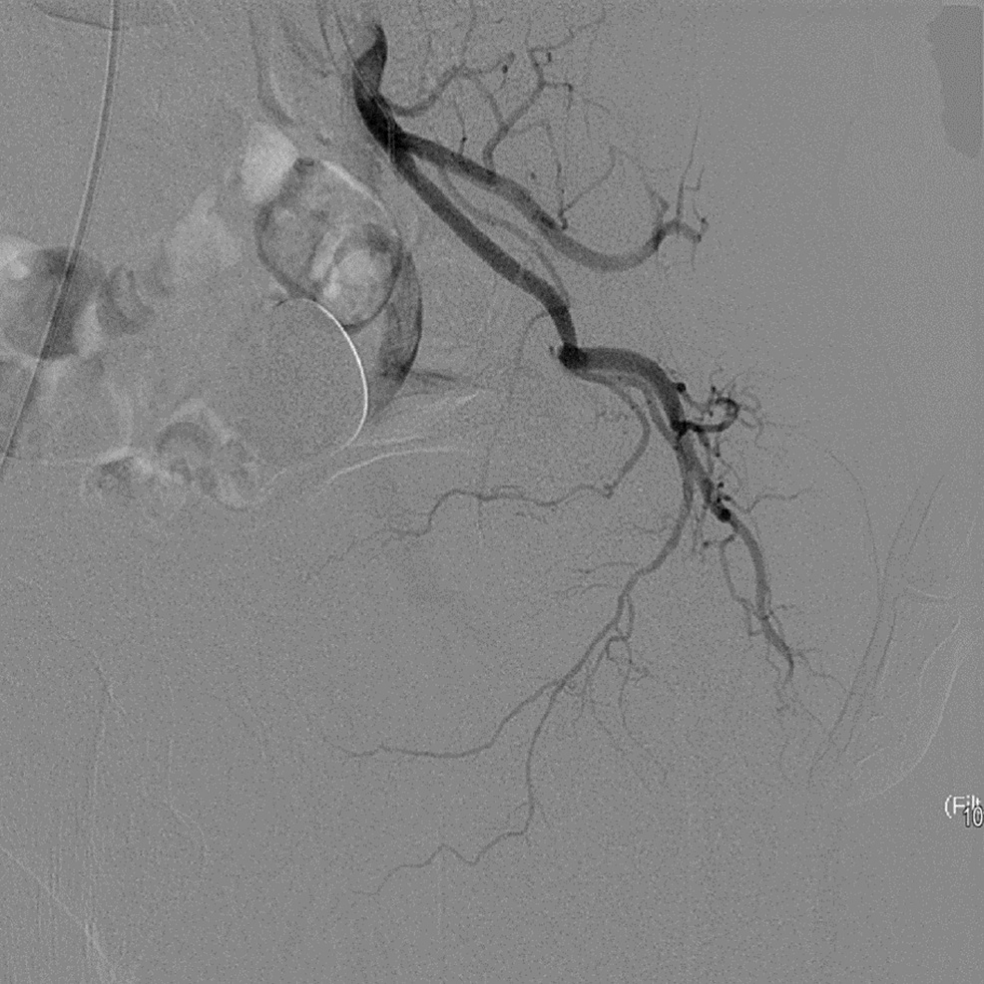
Pelvic angiogram by 5 Fr catheter in the left internal iliac artery showing common trunk of the prostatic artery and the superior vesical artery.

**Figure 3:**
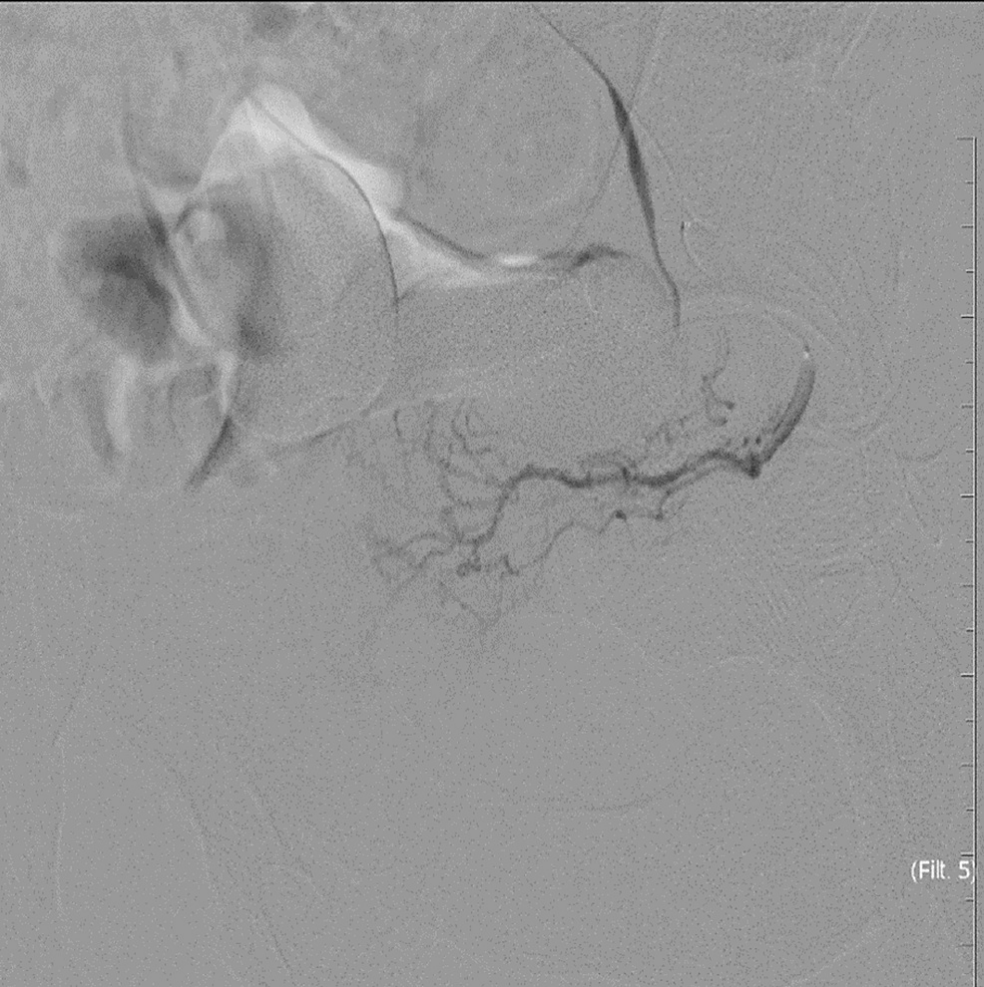
Selective angiogram of the left prostatic artery showing normal prostate blush.

**Figure 4:**
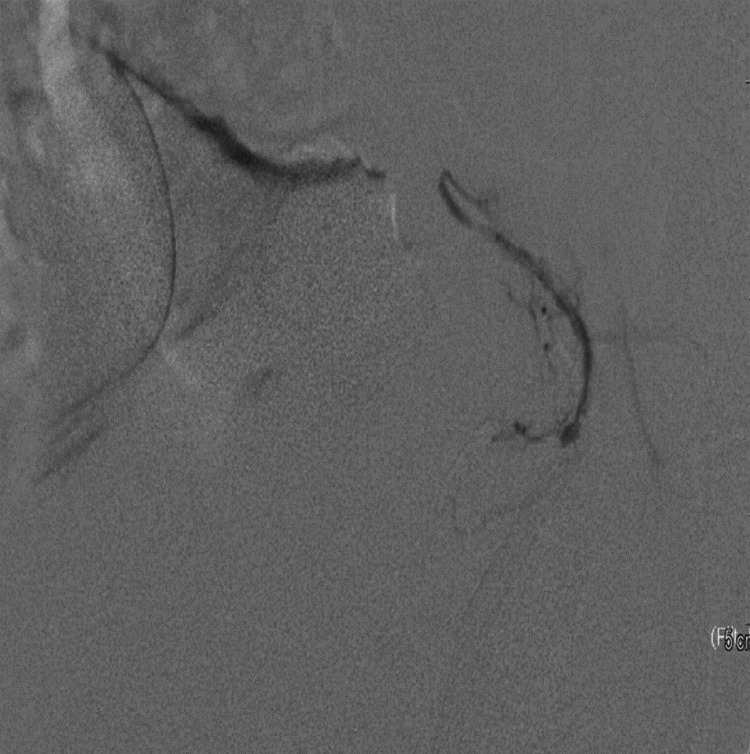
Post-embolization angiogram of the left prostate artery in the same patient showing no opacification of the prostate blush.

Embolization using 300-500 mic tris-acryl gelatin microspheres (vial of Embosphere 300-500 mic, Merit Medical, Salt Lake City, UT) was used. The catheter was retracted and redirected at the distal abdominal aorta followed by selective cannulation of the right prostatic artery. Dynamic CT was done again, confirming the position followed by embolization by the same types of microspheres. Post-embolization pelvic angiogram was done to confirm complete embolization of both prostatic arteries (Figures 2-4).

Patients were observed for 24 hours to detect any development of complications. PSA was measured 24 hours post-procedure. Patients were prescribed with ciprofloxacin antibiotic 500 mg twice daily for two days before and one week after the procedure, ibuprofen 400 mg twice daily for three days, and omeprazole 20 mg once daily post-procedure.

The post-procedure evaluation was performed at one, three, six, and 12 months. One and three months follow-up included IPSS, QOL, and IIEF questionnaires and PSA test. Six and 12 months follow up included the IIEF, IPSS, and QOL questionnaires, PSA test, MRI prostate, and flowmetry.

Outcome measure

Technical success was defined as at least unilateral PAE. Clinical success was defined as a decrease of IPSS score ≥ 3 points, QoL score ≤ 3 or a decrease by three points, and no need for surgical intervention.

Statistical analysis

Descriptive statistics were used to compute frequency distributions, mean, standard deviation, and ranges. The difference in continuous variables was evaluated by Student’s t-test. Calculations were performed using SPSS version 25.0 (IBM Corp., Armonk, NY). Results were considered statistically significant at p < 0.05.

## Results

Between June 2015 and August 2018, a total of 29 patients were included and prospectively followed up in this study. The mean age of the patients was 64.3 ± 8.1 years (range: 48-85 years). None of our patients was dependent on a catheter. Baseline patient characteristics are presented in Table 1.

**Table 1:** Baseline patient characteristics. PV = prostate volume; IPSS = International Prostate Symptom Score; QoL = quality of life; IIEF = International Index of Erectile Dysfunction; Qmax = maximal urine flow rate; PSA = prostate-specific antigen.

	Mean	SD	Range
Age	64.3	8.1	48-85
PV (ml)	87.6	40.1	40-206
IPSS	22.0	6.0	9-32
QoL	4.5	0.9	3-5
IIEF	13.4	10.3	0-26
Qmax (cm^3^/min)	18.6	27.2	2.5-28.8
PSA (ng/cm^3^)	2.8	1.9	0.6-5.0

The indication for the procedure was a failure of medical therapy and refusal of surgical treatment by the patient or due to the presence of multiple comorbidities increasing the risk associated with surgical intervention. Ten (34%) patients were referred from the urology clinic, while the rest of the patients were self-referred or were referred from other specialties. The procedure was performed by unilateral approach in all patients. Technical success was achieved for all patients. All patients, except for three, underwent bilateral PAE. Three patients underwent unilateral PAE because of severe atherosclerotic changes and tortuosity of the iliac and prostatic arteries. The procedure was performed in one session, except for one patient who complained of back pain, and consecutively, the procedure was rescheduled a month later. All patients were discharged after 24 hours. One patient experienced urinary retention and required catheterization for three days and was discharged after a successful trial of urination. The same patient never experienced urinary retention pre-operation or during the 12 months of follow-up. No patients encountered major complications.

**Figure 5:**
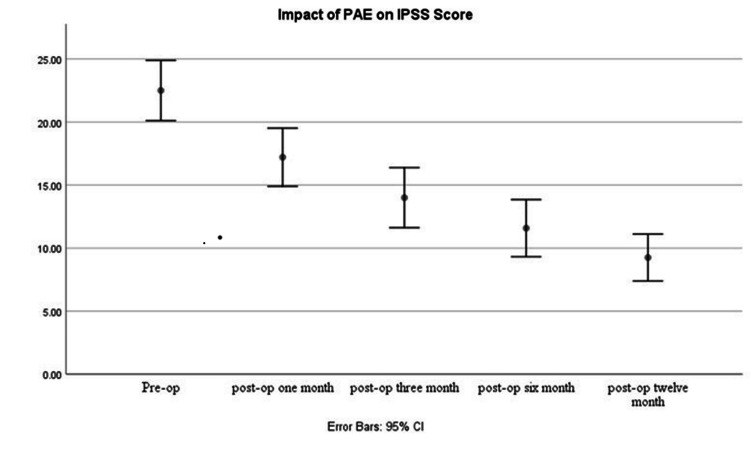
Graph showing a decrease of mean International Prostate Symptom Score (IPSS) over 12 months follow-up. PAE = prostatic artery embolization.

The mean IPSS score prior to the procedure was 21.96 (95% CI: 19.42-24.50). Data at six months showed a decrease of mean IPSS score to 11.44 (95% CI: 9.25-13.63, p < 0.01). Further decrease of mean IPSS score to 9.25 (95% CI: 7.39-11.11, p < 0.01) was noted at 12 months follow-up (Figure 5).

A significant decrease in PSA was seen during follow up after six months (mean difference: 10.52, 95% CI: 8.29-12.74, p < 0.01) and after 12 months (mean difference: 13.25, 95% CI: 11.01-15.48, p < 0.01). Pre-operative voiding symptom scores were significantly more than pre-operative storage symptom scores with a mean difference of 4.25 ± 2.13 (p = 0.005). Postoperatively, the scores significantly improved for storage (mean difference: 4.54 ± 2.93, p < 0.005) and voiding symptoms (mean difference: 7.54 ± 4.74, p < 0.005) (Table 2). There was no significant difference between storage and voiding symptoms scores postoperatively. However, voiding symptoms improved to a greater extent compared to storage symptoms. QoL score showed a mean difference of 2.2 ± 0.2 (p < 0.001) at 12 months follow-up (Table 2).

**Table 2:** Patient characteristics. PV = prostate volume; IPSS = International Prostate Symptom Score; QoL = quality of life; IIEF = International Index of Erectile Dysfunction; Qmax = maximal urine flow rate; PSA = prostate-specific antigen.

Variable and time point	Mean difference from baseline	95% CI
IPSS		
1 month	−6.04 ± 3.74	−8.1 to −4.6
3 month	−10.69 ± 4.23	−13.2 to −8.1
6 month	−11.80 ± 5.22	−14.6 to −8.9
12 month	−13.31 ± 5.56	−15.9 to −10.6
QoL score		
1 month	−1.41 ± 1.17	−2.0 to −0.8
3 month	−2.3. ± 0.77	−2.8 to −1.8
6 month	−2.4 ± 1.05	−2.9 to −1.8
12 month	−2.2 ± 1.29	−2.7 to 1.7
Qmax		
6 month	5.47 ± 10.24	0.19 to 11.14
12 month	6.83 ± 12.36	0.63 to 14.30
PV		
6 month	−39. 14 ± 39.00	−60.0 to −17.5
12 month	−39.13 ± 35.23	−57.2 to −21.0
PSA		
1 month	−0.77 ± 1.65	−1.68 to 0.14
3 month	−1.88 ± 1.94	−3.00 to −0.76
6 month	−1.38 ± 1.94	−2.39 to −0.37
12 month	−1.23 ± 1.71	−1.88 to −0.57
IIEF		
1 month	−0.38 ± 3.58	−2.17 to −1.39
3 month	−1.23 ± 3.41	−3.29 to −0.83
6 month	−0.66 ± 3.22	−2.71 to −1.38
12 month	−0.15 ± 2.71	−1.46 to −1.14

A significant increase in maximal urine flow rate (Qmax) was seen during follow-up after six months (mean difference: 4.34, 95% CI: 2.42-6.36, p = 0.024). The mean pre-operative prostate volume (PV) was 89.01 (range: 55-206). Follow up by MRI showed a significant decrease in PV after six months (mean difference: 30.05, 95% CI: 16.25-43.86, p < 0.01) and after 12 months (mean difference: 33.13, 95% CI: 19.50-46.76, p < 0.01) (Figure 5). IIEF score remained unchanged in all patients, except for one who had an increase of four points by 12 months of follow-up. A decrease in mean PSA level was seen during follow-up after three months (mean difference: 1.07, 95% CI: 0.26-1.37) and at six months (mean difference: 1.10, 95% CI: 0.36-1.84, p < 0.01).

Clinical success was achieved for 26 patients (89.6%). One of the three remaining patients had a recurrence of his symptoms at 10 months and underwent TURP. This patient had unilateral embolization. The two other patients were not improved by the end of the 12-month period. These patients had a prostate size of 62 and 75 ml and both received bilateral PAE.

There is a strong significant positive correlation between operative prostrate volume and 24 hour PSA (r = 0.538, p = 0.003). There is no significant relation between operative 24 hour PSA (r = 0.176, p = 0.531) and reduction of PV at 12 months and reduction of IPSS score at 12 months (r = 0.062, p = 0.800).

## Discussion

PAE is a minimally invasive technique that proved to be a successful standard treatment of BPH, especially in patients refractory to medical treatment, refusing or unsuitable for surgical treatment. Since the initial report by Carnevale et al. in 2010 [4], there has been a growing literature supporting PAE as an effective treatment of BPH. We report the clinical success of 89.6%, higher compared to other published data, ranging from 82.4% to 85% [3,5-7]. The higher result can be explained by performing bilateral procedures, high initial IPSS voiding score compared to storage symptoms score, mean age of sample >60 years, and high pre-procedure PV. Those are key factors that determine the success of PAE. However, we did not include these factors in our inclusion criteria as we aimed to simulate real day-to-day clinical practice. For the three patients who did not improve, the second trial of PAE could have been successful.

We noticed an improvement in both voiding and storage symptoms as reflected by the IPSS score. A slightly better improvement in the voiding score compared to the storage score was observed like other published studies [8,9]. One of our patients has noticed an improvement in sexual function as reflected by the IIEF score. A possible explanation is that patients avoided the adverse effects of medical therapy of BPH, namely, retrograde ejaculation caused by alpha-adrenergic blockers [10]. Although, PAE should not be advertised as a treatment of sexual dysfunction.

We included patients of different ages without taking into consideration the probability of vascular tortuosity and atherosclerotic changes prior to the procedure. Many studies advocate vascular imaging to identify vascular occlusion that results in technical failure, and may also reduce procedure time by knowing the anatomy of the prostatic artery [5,6].

One of our patients experienced post-procedure acute urinary retention (3.4%). The glandular edema post embolization compromises the prostatic urethra leading to retention of urine. It is a common minor complication reported in about 8% of patients [5-7]. On the other hand, the most commonly reported minor complication is dysuria in up to 10.4% [3,5,6].

The most feared complications are those related to non-targeted embolization of the rectum, bladder, or penis. Major complications also include vesical artery dissection and persistent UTI, requiring admission. Most of these complications were managed conservatively [5-7]. A systematic review by Kuang et al. included 788 patients and reported a 0.4% rate of major complications, which reflects the safety of PAE [5]. The use of dynamic CT during the procedure in our experience prevented non-target embolization by carefully identifying the points of anastomoses prior to injecting the embolizing material.

A high 24 hours PSA was not correlated with a higher decrease in prostate volume or IPSS score. On the contrary, Bilhim et al. found a positive correlation between the level of 24 hours PSA, decrease in IPSS, and ischemic volume [11].

Most centers perform PAE as an outpatient procedure (<24 hours) [6,7]. While we could have done this as an outpatient procedure, we opted to admit patients for one day to observe for complications. However, future procedures will be performed on an outpatient basis. We wanted to avoid the anxiety of patients and staff regarding this newly introduced intervention. As PAE is new in the country, we faced hesitancy from many patients and referring clinicians included. A comprehensive pre-operative consultation to explain to the patient what to expect from the procedure is an extremely important factor for the success of this intervention [12]. Nevertheless, the education of physicians regarding the role of PAE in managing BPH is equally important. Our study is limited by the small number of the population and the need for a longer follow-up period.

## Conclusions

Our experience, although limited, has proved so far that PAE is a promising, safe, non-invasive procedure for the treatment of BPH. We believe that PAE should be performed as an outpatient procedure. Patient and physician education about the advantages of PAE is needed. We add to the growing supporting literature of PAE by our initial single-center study from the Middle East.
